# Model Locomotives as a Garden Hobby

**Published:** 1913-08-30

**Authors:** 


### RELAXATIONS AND HOBBIES.

(Contributions are Invited)

### Model Locomotives as a Garden Hobby.

I was induced to take up model "locos" as a hobby through seeing a friend's miniature electric track worked from his consulting-room chair. The ease of manipulation, the cleanliness, and the interest of the thing came as a revelation; and, although I have no "mechanical kink," I felt that here was an almost ideal recreation for a practitioner who had scanty leisure and desired a pastime that should not be dependent upon the weather nor be devoid of a certain amount of intellectual pleasure without at the same time being too strenuous. My experience may be of use to others similarly situated.

I started with a Number 0 gauge (1¼ inches) electrically-driven tank "loco" of the London and North Western type. This was a handy compact little model, made to scale, and a perfect beauty in appearance. It needed a four to eight voltage, and with rolling-stock and sixteen-feet track complete cost me about three guineas. It is still working well, and has given endless pleasure to myself and to numerous friends and acquaintances whose minds are sufficiently childlike to appreciate the delights of miniature "locos." But I soon found that with practice there developed a desire to embark on more ambitious schemes. The disadvantage of electrically-driven engines is that they are not quite realistic enough; they cannot whistle and puff steam, and they need quite as much attention as, if not more than, the steam-driven "locos." Their great advantage is that they can be so easily and cleanly manipulated. With a switchboard it is possible to control all the movements of the train from one's table. Even accumulators may be dispensed with and the trains driven from the main, the track being connected by wire with the house current through a suitable switch or interposing apparatus. A complete electric installation makes an excellent outdoor track when accumulators are used; by carrying an accumulator in the tender of the engine or in a truck, and adapting the engine, the train can be run on an ordinary track without the use of a central rail. But for complete satisfaction the steam-driven engine stands supreme for getting realistic effects.

My initial attempt was with a small 0-gauge engine, purchased for half-a-guinea, which ran comfortably on the old electric track. A 1-gauge engine seemed preferable, and to avoid duplicating lines and stock I invested in engines of this gauge, which proves, all things considered, to be the most suitable for work on a small scale. The larger gauges are undoubtedly more impressive, but they require much larger rolling-stock and engines, and are altogther more expensive, while they can hardly be considered as toys. For one who is a deft and expert mechanic and understands the use of pliers and lathe, the larger gauges are admirable. But I have contented myself with the 1-gauge, which I have found generally satisfactory.

For one who is not expert as a mechanic it is advisable to buy the engines and rolling-stock ready made, or at least with all the castings prepared for fitting. Messrs. Bassett-Lowke, cf Northampton, who are the premier model-engine makers, supply an excellent model for those who wish to fit an engine together from "lathed castings"—that is to say, from parts already finished which need only assembling. This is their No. 1,000 Midland Express of the "Deeley" type, which is listed at four guineas, and is an admirable model for all ordinary work with the one inch and three-quarters gauge. Rolling-stock and track for such an engine come to about three guineas extra, although the expense in regard to these almost necessary adjuncts is limited only by the ambitions of the hobbyist. For an ordinary room a twelve-feet oval track, with curves and cross-overs, is perhaps the most suitable. The same firm supplies all necessary adjuncts in steel rails, tinplate rails, and trucks and carriages. A very fine and altogether satisfactory model listed is the No. 1,442 "Atlantic" type of the Great Northern Railway, priced at £5 10s. A very handsome one is the "Black Prince" type of the London and North Western Railway, which is a pound cheaper. More expensive types are the fine models, built to scale and perfect representations of the original, of foreign types of engines. In fact there is such a wide choice that the tyro has some difficulty in making a selection, but for obvious reasons it is preferable to start with a cheaper grade model.

For general pleasure and interest, model "locos" as a hobby would be hard to beat. Everyone has some interest in things that can be made to move. The delight of manipulating the engine increases when one comes to realise the immense possibilities in the way of variation that a model engine holds out. Nor is this all. There is a similar gratification in engineering a track through a back garden, overcoming obstacles and impediments, and establishing a smooth, level running track with bridges, culverts, stations, crossings, signals, and sidings. With the old 0-gauge electric track as extra line, a most realistic railway has been planned in my back yard, since it is too large for indoor use. This track is, of course, movable and can be taken up after use and readily laid down with a little trouble, half an hour's work being sufficient to lay the track, start the engines, and have everything ready for the train service. By arranging a small accumulator in the carriages and suitably adjusting wires to various parts, the train can be electrically lighted up, pea lamps being used on the engine and guard's van. Similarly, the stations and signals can be electrically lighted, the effect so obtained being most realistic. The expenditure of bodily energy is small in comparison with the amount of pleasure extracted from such a hobby; the expense entailed is small in comparison with that of certain other hobbies, such as, for example, golf.

